# Discrimination between human populations using a small number of differentially methylated CpG sites: a preliminary study using lymphoblastoid cell lines and peripheral blood samples of European and Chinese origin

**DOI:** 10.1186/s12864-020-07092-x

**Published:** 2020-10-12

**Authors:** Patrycja Daca-Roszak, Roman Jaksik, Julia Paczkowska, Michał Witt, Ewa Ziętkiewicz

**Affiliations:** 1 Institute of Human Genetics, Polish Academy of Sciences, Strzeszynska 32, 60-479 Poznan, Poland; 2 Silesian University of Technology, Akademicka 16, 44-100 Gliwice, Poland

**Keywords:** DNA methylation, Human population identification, Pyrosequencing, Population differentiating CpGs

## Abstract

**Background:**

Epigenetics is one of the factors shaping natural variability observed among human populations. A small proportion of heritable inter-population differences are observed in the context of both the genome-wide methylation level and the methylation status of individual CpG sites. It has been demonstrated that a limited number of carefully selected differentially methylated sites may allow discrimination between main human populations. However, most of the few published results have been performed exclusively on B-lymphocyte cell lines.

**Results:**

The goal of our study was to identify a set of CpG sites sufficient to discriminate between populations of European and Chinese ancestry based on the difference in the DNA methylation profile not only in cell lines but also in primary cell samples. The preliminary selection of CpG sites differentially methylated in these two populations (pop-CpGs) was based on the analysis of two groups of commercially available ethnically-specific B-lymphocyte cell lines, performed using *Illumina Infinium Human Methylation 450 BeadChip Array.* A subset of 10 pop-CpGs characterized by the best differentiating criteria (|Mdiff| > 1, q < 0.05; lack of the confounding genomic features), and 10 additional CpGs in their immediate vicinity, were further tested using pyrosequencing technology in both B-lymphocyte cell lines and in the primary samples of the peripheral blood representing two analyzed populations. To assess the population-discriminating potential of the selected set of CpGs (further referred to as “composite pop (CEU-CHB)-CpG marker”)*,* three classification methods were applied. The predictive ability of the composite 8-site pop (CEU-CHB)-CpG marker was assessed using 10-fold cross-validation method on two independent sets of samples.

**Conclusions:**

Our results showed that less than 10 pop-CpG sites may distinguish populations of European and Chinese ancestry; importantly, this small composite pop-CpG marker performs well in both lymphoblastoid cell lines and in non-homogenous blood samples regardless of a gender.

## Background

Genetic variation of human populations is extensively explored in a variety of fields including epidemiological and medical studies (e.g. population-specific susceptibility to diseases, pharmacogenomics), but also in evolutionary studies and forensics (e.g. populations origin, relationships, identification) [1–5]. The relation between the genome variation and population ancestry has been admittedly proven [6–9]. A variety of genomic markers (SNPs, CNVs, microsatellites, and mtDNA, Y-chromosome haplotypes) providing accurate ancestry information have been identified, validated and successfully implanted in population-stratification tests (e.g. [10–12]).

The differences between human populations are shaped not only by the genomic DNA variation but also by transcriptomic and DNA methylation variation [13–22]. Therefore, besides the most frequently used genomic DNA markers, some “non-classical markers”, representing inter-population differences in the expression and in the DNA methylation level, can potentially be used to discriminate between populations. In fact, a number of population-specific mRNA markers have been identified and tested in both B-cell lines and in a primary biological material, e.g. blood see [23].

It is well known that the majority of differences in the level of DNA methylation are caused by multiple environmental factors e.g. nutrition, exposure to pollutants, social conditions, etc. [24–27]. However, the recent development of high-throughput methods (mainly microarray technology) provided a wealth of data, which have demonstrated that a considerable part of the methylation variance reflects stable and heritable differences [28, 29]. Some of them are inter-individual and some differentiate populations [13, 18–20, 30–32]. The inter-population differences are observed in both the genome-wide methylation level and in the methylation status of individual CpG sites [15, 16, 19, 20, 33–35]. Compared to the genomic DNA variation, the persistent inter-population differences in the methylation level are rather small; nevertheless, they represent a possible source of markers that could be used for human population stratification. The inter-population differences in the level of methylation have been demonstrated in distinct types of a biological material: B-lymphocyte cell lines (e.g. [19, 20, 36, 37]), skin cells (e.g. [38, 39]), blood samples (e.g. [13, 30]). Moreover, it has been shown that even a limited number (~ 400 CpGs) of carefully selected differentially methylated CpG sites may allow discrimination of three main human groups: Americans of African origin, Europeans and Asians [20].

**Fig. 1:**
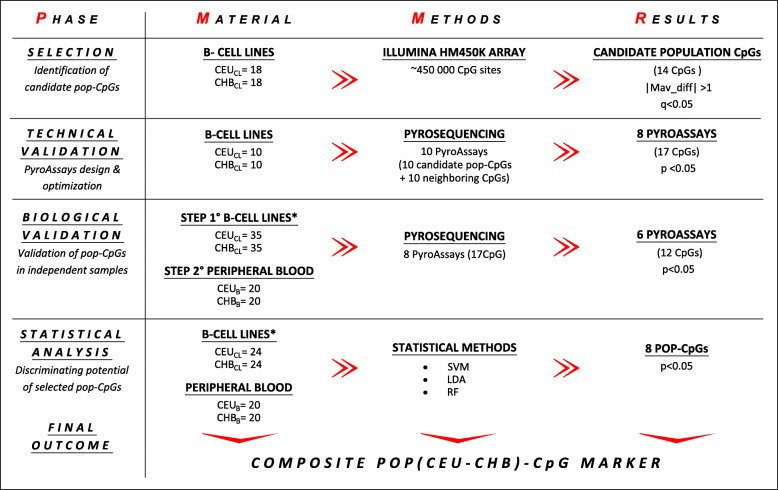
Study design. * cell lines other than those used in Illumina study. Authors’ original figure

The goal of our study was to identify a small set of differentially methylated CpG sites (pop-CpGs) sufficient to discriminate between populations of European and Chinese ancestry, which could be used as an easily manageable, composite pop (CEU-CHB)-CpG marker for a forensic differentiation between samples based on their population origin (see Fig. 1).

A set of 14 CpG sites characterized by significant population differences in their methylation (|Mdiff| > 1 at q < 0.05, and the lack of confounding SNPs under Illumina probes) was identified, based on the analysis of 36 commercially available B-lymphocyte cell lines of European and Chinese origin, performed using *Illumina Infinium Human Methylation 450 BeadChip Array*. A subset of 10 CpGs characterized by the best criteria, and 10 additional CpGs in their immediate vicinity, was further tested in both B-lymphocyte cell lines and in primary samples of peripheral blood. Statistical evaluation of the discriminating potential of the best-performing pop-CpGs, employing 10-fold cross-validation method, was then performed in two independent sets of samples.

## Results

### Selection of candidate pop-CpGs

*Illumina Infinium HumanMethylation 450 BeadChip Array* (HM450K array), previously applied to characterize methylation level in B-lymphocyte cell lines representing CEU (*n* = 18) and CHB (*n* = 18), revealed a set of 96 CpGs, differentiating the two populations at the significance level *p* < 0.05, and representing the highest inter-population differences in the average methylation levels (|M_av__diff| > 1; q < 0.05) see [40]. From these differentially methylated CpGs, a small set of 14, characterized by the absence of confounding features (lack of SNPs in the studied CpG, lack of frequent SNPs under Illumina probe; no multi-site mapping of the probe), was selected as candidate pop-CpGs (Table 1).

**Table 1:** Characteristics of the candidate pop-CpGs

nb	Candidate pop- CpGs	Genomic position (GRCh:37)	Locus	Gene region	Type of region	|M_**av**__diff|	q-value
1	**cg18136963**	chr6:139013146	*FLJ49*	not provided	N_Shore	2.950	0.0355
2	**cg26367031**	chr3:178984747	*KCNMB3*	5’UTR; 1st exon	not provided	2.775	0.0215
3	**cg03140118**	chr1:37939320	*ZC3H12A*	TSS1500	N_Shore	2.411	0.001
4	**cg23669876**	chr1: 36489276	*AGO3*	Body (LTR)	not provided	2.355	0.0039
5	**cg00862290**	chr3:178984973	*KCNMB3*	TSS200	S_Shore	2.247	0.008
6	**cg08979191**	chr5:132113734	*SEPT8*	TSS200	S_Shore	1.875	0.0185
7	cg24037715	chr14: 35203968	–	not provided	sea	1.691	0.0003
8	**cg07207043**	chr6:7051497	*RREB1*	not provided	CpG Island	1.534	0.0345
9	**cg04036182**	chr15:45458818	*SHF*	not provided	CpG Island	1.451	0.0201
10	cg00031303	chr3: 195681400	SDHAP1	not provided	CpG Island	1.359	0.005
11	**cg07904028**	chr4:6328508	*PPP2R2C*	body	not provided	1.257	0.0145
12	cg09972454	chr16: 15083088	*PDXDC1*	body	N_Shore	1.232	0.0029
13	**cg24861686**	chr8:11418058	*BLK*	body	N_Shelf	1.193	0.000
14	cg03585734	chr1: 15598865	*FHAD1*	body	not provided	1.123	0.0144

CpGs selected for pyrosequencing validation are bolded. Shores and shelves are defined in Illumina as regions 0–2 kb and 2–4 kb, respectively, from a CpG island. *N* Upstream, *S* Downstream, *TSS* Transcription site start, *LTR* Long terminal region

Eleven of 14 best-differentiating CpGs were located outside CpG islands (in shore or shelf regions, gene body, transcription site start or 5’UTR regions). Three CpG sites*,* cg04036182 (chr15:45458818), cg07207043 (chr6:7051497) and cg00031303 (chr3: 195681400), were located in the genomic island of *SHF*, *RREB1* and SDHAP1 genes, respectively. The highest inter-population differences in the methylation level (~ 40% difference) were observed in cg18136963 (chr6:139013146) and cg26367031 (chr3:178984747) (M_av__diff ≥2.7).

#### DNA methylation and gene expression correlation analysis

Thirty-six B-lymphocyte cell lines from both populations (CEU and CHB) were analyzed on HM450 array (Illumina) and HumanHT-12v4 Expression BeadChip Kit expression array (Illumina). Based on the results obtained from both Illumina platforms, a t-test was performed to identify CpG loci and genes, showing statistically significant inter-population differences in the level of DNA methylation and in the gene expression, respectively. Subsequently, to identify a relation between the gene expression and the corresponding methylation status, a Pearson correlation analysis was performed.

Based on the two-step statistical analysis, a group of genes and CpG loci meeting statistical criteria, *p* < 0.01 in t-tests and in Pearson correlation analysis, was identified. None of the pop-CpGs, except for cg24861686 (1_CpG1, chr8:11418058), met the abovementioned statistical criteria. This CpG site showed positive correlation with *BLK* gene (Pearson coefficient 0.63).

### Technical validation

A subset of 10 pop-CpGs candidates meeting even more stringent statistical criteria (|M_av__diff| ≥ 1.2 at q < 0.05), and 10 additional CpGs located in their close proximity, was analyzed using pyrosequencing technique (Table 2).

**Table 2:** Comparison of DNA methylation levels assessed using Illumina HM450K array and pyrosequencing assays (PyroAssays)

CpG name in HM450K array	PyroAssay name	Illumina infinium human methylation 450BEAD chip array				Pyrosequencing technical validation			
		beta_mean_CEU	beta_mean_CHB	CEU.beta_mean -CHB.beta_mean	q- value	CEU.mean	CHB.mean	CEU.mean -CHB.mean	***p***-value_beta
		(***n*** = 18)	(***n*** = 18)			(***n*** = 10)	(***n*** = 10)		
**cg24861686**	1_CpG1^a^	0.841	0.697	0.143	0.0000	0.813	0.591	0.222	0.0000
**cg03140118**	2_CpG1^a^	0.176	0.503	−0.327	0.0010	0.131	0.518	−0.387	0.0003
	3_CpG1	–	–	–	–	0.410	0.150	0.259	0.0056
	3_CpG2	–	–	–	–	0.289	0.087	0.202	0.0048
**cg00862290**	3_CpG3^a^	0.466	0.161	0.305	0.0080	–	–	–	–
**cg07904028**	4_CpG1^a^	0.515	0.714	−0.199	0.0145	–	–	–	–
**cg08979191**	5_CpG1^a^	0.779	0.520	0.258	0.0185	0.782	0.544	0.238	0.0117
	5_CpG2	–	–	–	–	0.609	0.400	0.209	0.1174
	5_CpG3	–	–	–	–	0.599	0.418	0.181	0.1942
	6_CpG1	–	–	–	–	0.067	0.186	−0.119	0.0106
**cg04036182**	6_CpG2^a^	0.271	0.486	−0.215	0.0201	0.112	0.470	−0.358	0.0000
**cg26367031**	7_CpG1^a^	0.539	0.170	0.369	0.0215	–	–	–	–
**cg18136963**	8_CpG1	–	–	–	–	0.520	0.179	0.341	0.0019
	8_CpG2^a^	0.514	0.162	0.352	0.0355	0.498	0.174	0.324	0.0097
	8_CpG3	–	–	–	–	0.423	0.179	0.243	0.0180
**cg07207043**	9_CpG1^a^	0.625	0.820	−0.195	0.0345	0.529	0.813	−0.283	0.0023
	9_CpG2	–	–	–	–	0.422	0.726	−0.304	0.0007
	9_CpG3	–	–	–	–	0.480	0.814	−0.335	0.0004
	10_CpG1	–	–	–	–	0.258	0.531	−0.272	0.0000
**cg23669876**	10_CpG2^a^	0.368	0.728	−0.360		0.290	0.590	−0.299	0.0000

HM450K array results are available only for HM450K-based candidate pop-CpGs (marked with ^a^). For cg00862290, which corresponds to the third CpG locus in PyroAssay 3, no reliable pyrosequencing data was obtained. Assays 4 (cg07904028) and 7 (cg26367031) did not pass technical evaluation step

Due to technical reason (see Additional file 1 for details), some CpGs were excluded, and a subset of 17 CpGs was analyzed in further experiments.

**Fig. 2:**
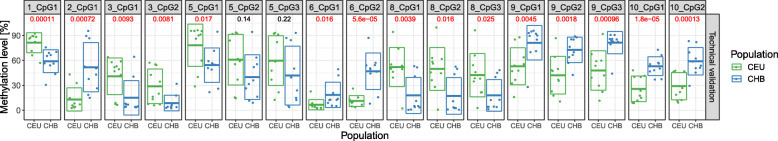
Results of the technical validation of eight PyroAssays. Twenty B-lymphocyte cell lines (10 from each population) were tested. The originally selected candidate pop-CpGs targeted in each PyroAssay are marked with *. Green – CEU population; blue – CHB population. Dots represent methylation levels in individual samples. Box plots denote mean value (lines inside the boxes) and standard deviation. Statistically significant (*p* < 0.05) population differences in the methylation level are marked in red

Pyrosequencing results were collected as proportional values, separately for each analyzed CpG site (Table 2, Fig. 2). The average value of differences in methylation level between the studied populations was in the range of 0.119 (PyroAssay 6_CpG1 chr15:45458826) to 0.387 (PyroAssay 2_CpG1 chr1:37939320). Statistically significant population differences (*p* < 0.05) were obtained for most of the CpG sites. The results from pyrosequencing were concordant with the results from HM450K array. The only exception was PyroAssay 5, where no statistically significant population differences in the level of methylation were noted for two out of the three examined CpGs (5_CpG2 chr5:132113755 and 5_CpG3 chr5:132113777); nevertheless, this PyroAssay was not excluded from further analyzes.

Figure 2 shows the distribution of methylation levels in individual B-lymphocyte cell lines used in the technical validation phase. Eight PyroAssays (1, 2, 3, 5, 6, 8, 9 and 10) passed the technical validation and were used in the further step of biological validation.

### Biological validation of population differences in methylation level

#### Independent B-lymphocyte cell lines

To test the biological validity of population-differentiating methylation status of 17 CpG sites, eight PyroAssays were performed in the independent set of B-lymphocyte cell lines. Statistically significant (*p* < 0.05) population differences in the mean methylation level were observed for 6 out of 8 tested PyroAssays (covering 12 CpG sites, see Table 3).

**Table 3:** Validation of eight PyroAssays performed in the independent set of B-lymphocyte cell lines

PyroAssay number_ position of CpG in the assay	CEU (n)	CHB (n)	CEU.mean	CHB.mean	CEU.var	CHB.var	CEU.mean - CHB.mean	padj_beta	***Pop_diff*** potential
**1_CpG1**	34	34	0.800	0.759	0.008	0.006	0.040	0.032	1
2_CpG1	34	34	0.243	0.252	0.052	0.040	−0.008	0.723	0
3_CpG1	34	34	0.246	0.222	0.069	0.051	0.024	0.828	0
3_CpG2	34	34	0.203	0.168	0.044	0.031	0.035	0.696	0
**5_CpG1**	34	34	0.718	0.594	0.057	0.041	0.124	0.049	1
**5_CpG2**	34	34	0.561	0.420	0.046	0.046	0.141	0.040	1
5_CpG3	34	34	0.522	0.448	0.064	0.049	0.074	0.319	0
**6_CpG1**	34	34	0.132	0.242	0.017	0.029	−0.110	0.007	1
**6_CpG2**	34	34	0.236	0.343	0.036	0.031	−0.107	0.018	1
**8_CpG1**	35	35	0.481	0.180	0.111	0.039	0.301	0.000	1
**8_CpG2**	35	35	0.492	0.166	0.125	0.050	0.325	0.000	1
**8_CpG3**	35	35	0.459	0.193	0.108	0.050	0.267	0.002	1
9_CpG1	34	34	0.713	0.806	0.042	0.035	−0.093	0.075	0
**9_CpG2**	34	34	0.632	0.772	0.035	0.021	−0.140	0.001	1
**9_CpG3**	34	34	0.657	0.784	0.049	0.030	−0.127	0.017	1
**10_CpG1**	30	31	0.146	0.561	0.035	0.055	−0.415	0.000	1
**10_CpG2**	30	31	0.171	0.640	0.043	0.062	−0.469	0.000	1

CpG sites characterized by statistically significant inter-population differences in their methylation level are bolded. padj_beta: p-value after Benjamin Hochberg correction; pop-diff potential: differentiation potential of individual sites: 0-non-differentiating; 1-differentiating

In the majority of PyroAssays, the level of methylation was similar across the neighboring CpG sites (Table 3). Only two CpGs (5_CpG3 chr5:132113777 and 9_CpG1 chr6:7051497) had distinct methylation level compared to the rest of positions targeted by the respective PyroAssay, with no statistically significant differences between the two populations (Table 3). The highest inter-population differences in methylation level were noted for CpGs covered by PyroAssays 8 and 10 (Table 3, CEUmean-CHBmean column). PyroAssays 2 and 3 didn’t reveal any statistically significant population differences in CpG methylation.

#### Peripheral blood samples

To test, whether population differences in the methylation levels of CpGs observed in CEU and CHB cell lines, reflected real differences between the two populations (and were not due to the cell lines’ peculiarities), the second step of biological validation was performed, using a primary biological material, i.e. peripheral blood samples from individuals representing two analyzed populations (*n* = 40 from both CEU and CHB).

**Fig. 3:**
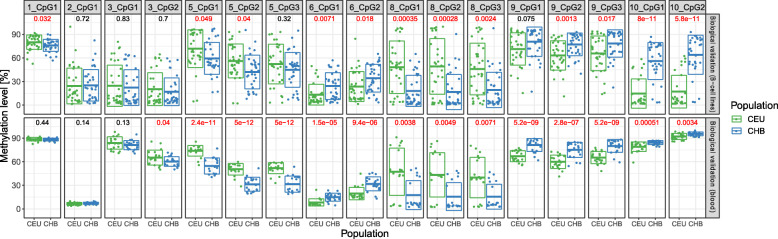
Biological validation of the methylation level at 12 CpG sites, performed in B-lymphocyte cell lines (upper panel) and blood samples (lower panel). Dots represent methylation level in the individual samples. Box plots denote mean value (lines inside the boxes) and standard deviation. Statistically significant (*p* < 0.05) population differences in the methylation level are marked in red

Overall, PyroAssays revealed similar inter-population differences in the level of CpG methylation in both B-lymphocyte cell lines and in blood samples. Furthermore, similar to the results obtained in B-lymphocyte cell lines, a high consistency in the methylation level among individual CpG sites examined within a given PyroAssay was also observed in blood samples (Fig. 3). The greatest inter-population differences in the level of CpG methylation was observed in PyroAssays 8 and 5. Only few inconsistencies were observed between B-lymphocyte cell lines and blood samples. Population differences in the methylation of 5_CpG3 (chr5:132113777) and 9_CpG1 (chr6:7051497) sites, which did not reach statistical significance in B-cell lines, were statistically significant in blood samples, whereas the inter-population differences in 1_CpG1 (chr8:11418058) were not significant in blood samples. On the other hand, CpG sites targeted by PyroAssay 10, which classified as strongly population-differentiating sites in the B-cell lines, in blood samples were characterized by the lowest average differences in their methylation values.

For the majority of PyroAssays, methylation readouts in individual blood samples were tightly clustered, as opposed to those observed in B-lymphocyte cell lines. The only exception was PyroAssay 8, where the spread of the readouts from blood samples was much larger, and had a clear a tri-modal methylation distribution (see Discussion).

### Discriminating potential of the selected pop-CpGs

#### Identification of a composite pop (CEU-CHB)-CpG marker

**Fig. 4:**
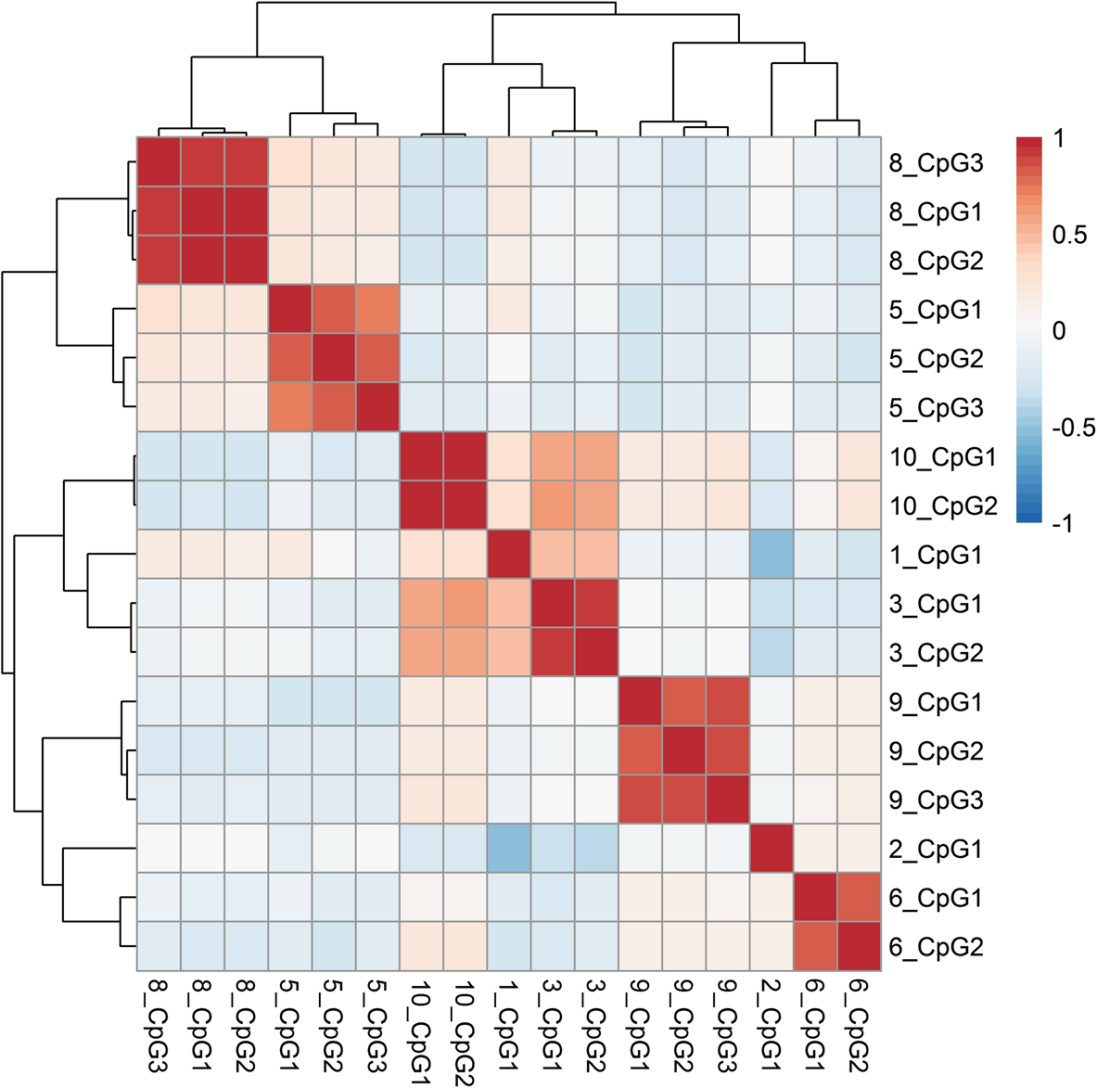
Correlation matrix showing the results of Pearson correlation analysis. Analysis was performed using data from PyroAssays performed in 20 B-lymphocyte cell lines (*n* = 10 from CEU, *n* = 10 from CHB population). Pearson correlation coefficient values and directions are marked with different colors; positive correlation (from white to red on the color scale); negative correlation (from white to blue) (see color-bar next to the matrix)

Pearson correlation analysis was performed using data from B-lymphocyte cell lines analysis (*n* = 10 CEU; *n* = 10 CHB) obtained during the technical validation step. Analysis showed a high correlation coefficient (0.8–1) within each of the corresponding PyroAssays, and simultaneously a low correlation (< 0.5) between individual PyroAssays (see Fig. 4 below).

To select the non-redundant set of validated pop-CpGs, correlated sites identified in the Pearson correlation analysis in each of the PyroAssays were removed. Based on the *p*-value after Benjamin Hochberg correction (the lowest padj_beta values were selected, see Table 3), a set of eight CpG sites (1_CpG1 chr8:11418058, 2_CpG1 chr1:37939320, 3_CpG2 chr3:178984959, 5_CpG1 chr5:132113734, 6_CpG2 chr15:45458818, 8_CpG1 chr6:139013142, 9_CpG3 chr6:7051504, 10_CpG1 chr1: 36489272) was selected. This set of eight non-redundant, validated pop-CpGs formed a composite pop (CEU-CHB)-CpG marker, with the potential to discriminate between CEU and CHB populations based on the differences in the level of methylation.

#### Testing of the composite pop (CEU-CHB)-CpG marker

To assess the population-discriminating potential of the 8-site composite pop (CEU-CHB)-CpG marker*,* three different classification methods were used: support vector machines (SVM) with linear kernel, linear discriminant analysis (LDA) and random forest (RF). The predictive ability of each method was assessed using 10-fold cross-validation, which was repeated 1000 times due to the moderate number of available cases.

**Fig. 5:**
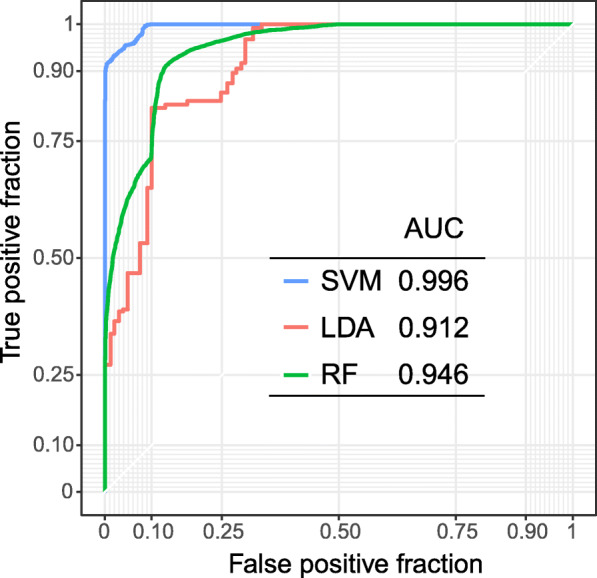
Accuracy of the classification using three different classification methods. A ROC curve and AUC parameter were calculated for: support vector machines (SVM; blue line), linear discriminate analysis LDA (red line), and random forest (RF; green line). Results were obtained based on B-lymphocyte cell lines (*n* = 20 from CEU and CHB). The ROC curve was created by plotting the true positive fraction against the false positive fraction at various threshold settings

The results obtained using each of the classification algorithms (SVM, LDA and RF) were compared in terms of AUC parameter (area under ROC curve) (see Fig. 5).

The shape of all presented curves followed the left-hand corner and the top border, indicating the high accuracy of the 8-site composite pop (CEU-CHB)-CpG marker with a high level of true positive in comparison to false positive results. Similar result was obtained using all three tested classification methods (AUC > 0.9), of which SVM was the most reliable (AUC = 0.996). The SVM validation performed on two independent datasets, B-lymphocyte cell lines (*n* = 48) and blood samples (*n* = 40), showed a high accuracy of the classification power in both sets (> 85%) (see Additional file 2).

**Fig. 6:**
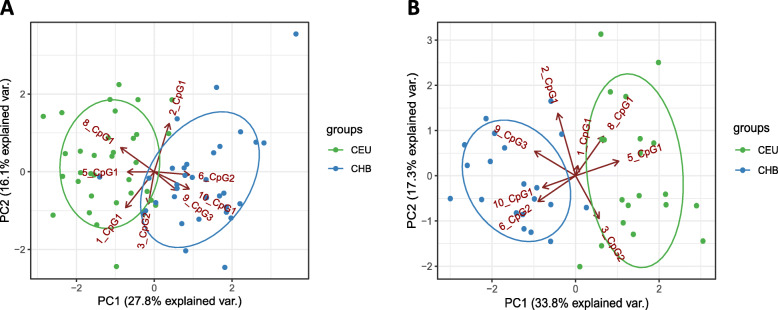
PC analysis separating samples from two populations. Analysis was performed on B-lymphocyte cell lines (*n* = 48 from CEU and CHB; **a** and blood samples (*n* = 40 from CEU and CHB; **b**. Vectors length denotes the power of influence of each feature on principal component, while vectors location show how variables correlate with one another. Dots represent individuals from CEU (green points) and CHB (blue points) population

Principle Component Analysis was used to assess the potential of the 8-site composite pop (CEU-CHB)-CpG marker to separate samples from two analyzed populations. While the vast majority of samples clustered according to their population affiliation, two population-specific clusters were located in the close vicinity. The more accurate separation was obtained for blood samples (population-specific clusters were more separated from each other compared to B-cell samples) (Fig. 6a, b).

The variance distribution was attributed to the first (~ 30%) and the second (~ 17%) dimension in both B-lymphocyte cell lines and blood samples. In both PC plots, markers 2_CpG1 (chr1:37939320, 6_CpG2 (chr15:45458818), 9_CpG3 (chr6:7051504) and 10_CpG1 (chr1:36489272) correlated with each other and showed higher methylation level in CHB population, whereas markers 1_CpG1 (chr8:11418058), 3_CpG2 (chr3:178984959), 8_CpG1 (chr6:139013142) and 5_CpG1 (chr5:132113734) showed higher metylation level in CEU population. The weight of an individual CpG marker on the principle component was diverse, as indicated by the vectors length. What is interesting, most CpG markers had similar weight in PC analyzed in B-lymphocyte cell lines (Fig. 6a), while in blood sample, the impact of one marker, 1_CpG1 (chr8:11418058), was distinctly smaller (Fig. 6b).

**Fig. 7:**
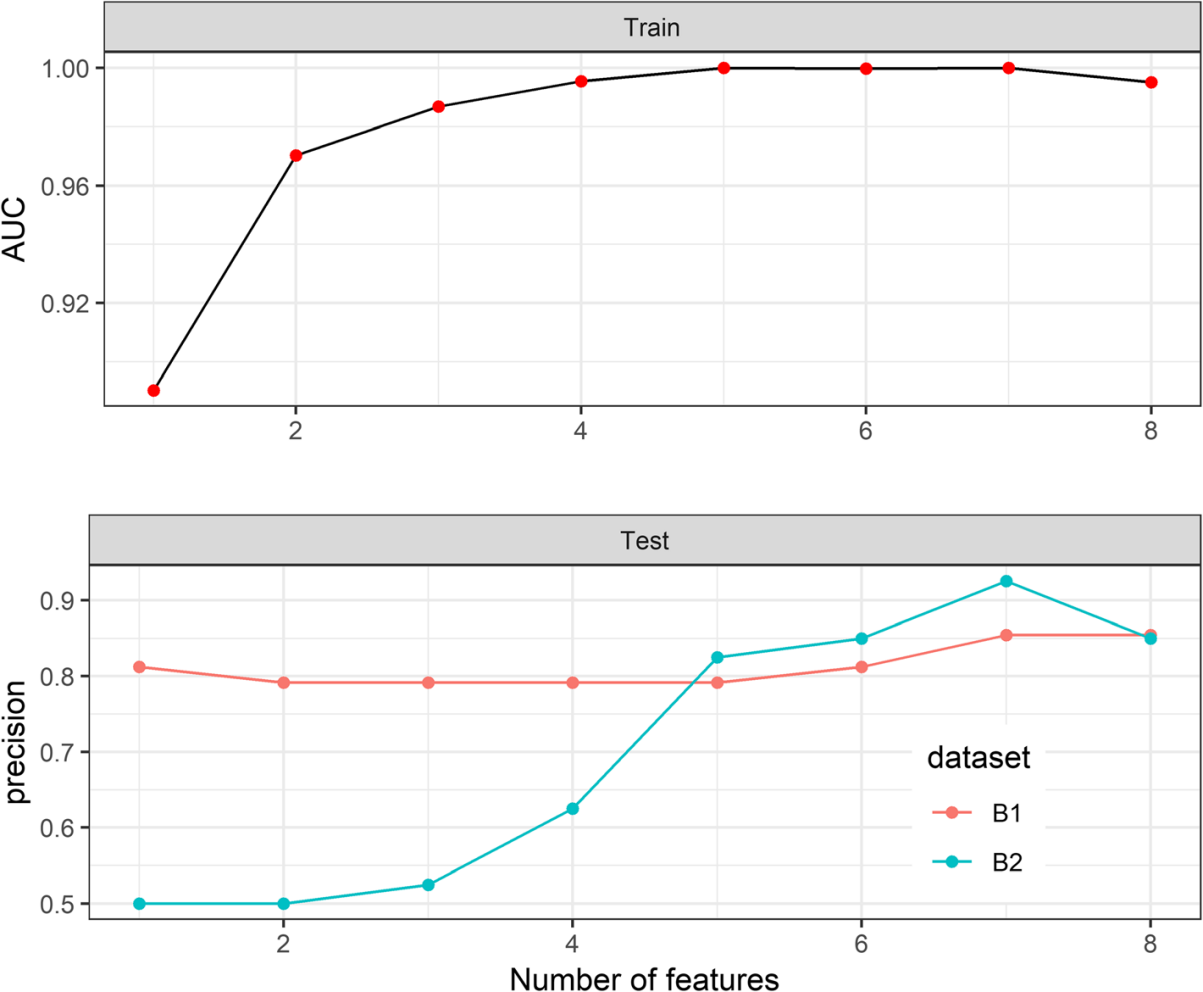
Relation between the number of CpG used in a training group and the quality of classification. Upper panel: The value of AUC parameter obtained in the training group (B-cell lines: CEU, *n* = 10; CHB, *n* = 10) depending on the number of CpG studied. Lower panel: Precision of sample classification depending on the number of features (CpGs) analyzed in two tested groups: B-lymphocyte cell lines (B1, red color, *n* = 48) and blood samples (B2, blue color, *n* = 40)

An additional test was performed to assess the minimal number of popCpGs that would classify individuals of European and Chinese ancestry with high accuracy. The minimal number of seven unlinked pop-CpGs (10_CpG1 chr1:36489272, 6_CpG2 chr15:45458818, 1_CpG1 chr8:11418058, 2_CpG1 chr1:37939320, 9_CpG3 chr6:7051504, 8_CpG1 chr6:139013142, 3_CpG2 chr3:178984959) had a high classification accuracy (AUC ~ 1, and precision> 0.8) (Fig. 7, lower panel) in both B-lymphocyte cell lines and blood samples; discrimination potential obtained in peripheral blood samples (precision =0.925) was higher in comparison to B-lymphocyte cell lines (precision = 0.854). In order to obtain similar discrimination power in both B-lymphocyte cell lines and peripheral blood samples, we decided to retain the 8-site composite pop (CEU-CHB)-CpG marker to be used for methylation-based classification of CEU and CHB populations (see Fig. 7, lower panel).

To assess the population-discriminating potential of the 8-site composite pop (CEU-CHB)-CpG marker on the individuals of both genders, an in silico analysis was performed using additional DNA methylation data for B-lymphocyte cell lines investigated on *Illumina Infinium Human Methylation 450 BeadChip Array* platform, obtained from GEO database (GSE36369). The SVM validation performed on two independent datasets: 93 Males (CEU = 47; CHB = 46) and 99 Females (CEU = 49; CHB = 50), showed a high accuracy of the classification power in both genders (> 89%) (see Additional file 3).

Furthermore, a biological validation of the 8-point composite pop (CEU-CHB)-CpG marker was performed. Male and Female blood samples from CEU (*n* = 96) and CHB (*n* = 96) population were obtained from the same Illumina microarray experiment as before (GSE36369). Results, similar to those coming from B-lymphocyte cell lines, indicated high population discrimination potential of our 8-point marker, regardless of the gender (see Additional file 4).

## Discussion

The aim of our study was to identify a set of CpG sites characterized by a significant difference in the DNA methylation profile between individuals of European and Chinese ancestry. Mainly adult males were analyzed. Analysis of 18 CEU and 18 CHB B-lymphocyte cell lines, performed on HM450K array, which measures the methylation of approximately 480,000 CpG sites across the human genome in parallel, revealed only 14 CpG sites with significantly different methylation levels in the studied populations (|M_av__diff| ≥ 1.0 and q < 0.05). According to the literature [41], |M_av__diff| ≥ 1.0 corresponds to a 20% difference in the methylation value. Such a small number of potentially population-differentiating CpG sites, with a relatively low inter-population differences in the methylation level (|M_diff_| in the range of 1.1–2.9), stands in line with results from other studies. It has been estimated that only a small fraction of CpGs across the genome stably varies in their methylation among human populations [18, 20, 42, 43].

A subset of 10 CpGs preselected in HM450K array experiment (cg24861686 chr8:11418058, cg03140118 chr1:37939320, cg00862290 chr3:178984973, cg07904028 chr4:6328508, cg08979191 chr5:132113734, cg04036182 chr15:45458818, cg26367031 chr3:178984747, cg18136963 chr6:139013146, cg07207043 chr6:7051497, cg23669876 chr1: 36489276), referred to as candidate pop-CpGs, with the highest inter-population differences in the mean methylation value (|M_av__diff| ≥ 1.2 and q-val < 0.05), was selected for further validation. Validation was done using pyrosequencing technique, regarded as a more sensitive method and widely used in DNA methylation studies [44, 45]. PyroAssays covered 10 candidate pop-CpGs, and several closely located neighboring CpGs, such that overall 20 CpG sites were tested.

Two-step validation was performed to exclude technical obstacles that could provide faulty results (technical validation in B-lymphocyte cell lines). In the next step, a biological validation in peripheral blood samples was performed to exclude the possibility that the inter-population differences in the methylation level reflected specific conditions related to the maintenance of the CHB and CEU cell lines. Following these two steps. Six out of the eight PyroAssays tested on primary material displayed statistically significant inter-population differences in the methylation level (*p* < 0.05).

These results indicated that the candidate population-differentiating CpG sites selected based on the analysis of B-lymphocyte cell lines, after a proper validation, may be used as population-differentiating markers also in the primary cells (blood samples) (see also [19]).

Our results are especially interesting in the context of a widely discussed suitability of B-lymphocyte cell lines (lymphoblastoid cell lines, LCLs) for population studies on methylation [46, 47]. LCLs are a commonly used source of biological material due to their easy availability (Coriell repository resources), tissue homogeneity (exclusively B-lymphocytes) and known population origin. However, some recent studies revealed that laboratory treatment of LCLs, e.g. EBV transformation or specific conditions during culturing (e.g. repeated freeze-thaw cycles), may induce random DNA methylation alterations and thus produce misleading methylation results [46–48]. In this context, a comparison of raw methylation readouts collected from B-lymphocyte cell lines and from blood samples in our study revealed interesting observations. A high consistency in the methylation level was observed among CpGs examined within each PyroAssay, both in B-lymphocyte cell lines and in blood samples. However, the mean values of inter-population differences in blood samples were smaller than in B-lymphocyte cell lines, and did not exceed 30% as opposed to nearly 50% in the cell lines. On the other hand, for the majority of CpGs, the readouts representing methylation in individual cell lines were scattered, while those representing individual blood samples remained “tightly” clustered around the mean (except for PyroAssay 8, see discussion below). The scattered methylation readouts observed in the cell lines could reflect the lack of homogeneity of technical (cell line maintenance etc.) and/or biological factors (age, and/or lifestyle of cell lines donors). Analysis of the reported age of B-lymphocyte cell lines donors (wherever available) revealed no correlation with the methylation results. Aspects related to the cell line maintenance were beyond our control (cells were purchased from Coriell Repository), but these lines have been used in many studies and to our knowledge no systemic population differences have been reported. The small variance of readouts observed in the primary biological material is more surprising. Knowing that blood is a mixture of different cell types, and that blood donors were not controlled for their lifestyle (e.g. diet, smoking etc.), methylation readouts were expected to be more scattered. On the other hand, the number of blood samples used in the analysis was lower than that of the cell lines, and it is possible that increasing the size of tested group would affect the picture.

The only exception from the generally small variance of the methylation readouts in blood samples was PyroAssay 8, where the distribution of readouts followed a characteristic tri-modal pattern. This pattern, when observed in HM450K array, has been described to reflect the presence of SNP in the examined CpG sites or in sequences targeted by Illumina probes (see [40, 49]). Although all PyroAssays in this study were designed to avoid SNP-related bias, a tri-modal pattern observed in PyroAssay 8 prompted us to subject it to a careful scrutiny, to exclude the possible impact of the genomic sequences. Both *in sillico* analysis, performed in Genome Browser Database, and Sanger sequencing of several B-lymphocyte cell lines and blood samples, did not reveal any SNPs/indels in either interrogated CpG sites and under the primers used in PyroAssays (data not shown). It is probable that, here also, increasing the number of samples could change this picture. In fact, an indication of a tri-modal distribution in PyroAssay 8 was also detectable in B-lymphocyte cell lines, but the larger number of samples blurred it into a cloudlike pattern (see Fig. 3).

To confirm the discriminating power of the composite pop (CEU-CHB)-CpG marker, composed of the validated pop-CpGs, a number of statistical analyzes were performed. All three algorithms (SVM, RF and LDA) used to test the sensitivity and specificity (ROC and AUC parameters) of population classification worked well in both types of the biological material (B-lymphocyte cell lines and blood samples), revealing high precision (> 90%) of sample population classification. What is more, our 8-point composite marker had a high population discrimination potential regardless of the gender, as shown by an in silico analysis of B-lymphocyte cell lines and blood samples.

According to the literature, a subset of population-specific methylation markers (< 500 pop-CpG sites) allows to carry out discrimination of main human populations. The set of eight pop-CpGs described in our study is, to our knowledge, the smallest methylation-based composite marker able to discriminate two human populations [13, 20, 43]. Principal component analysis using the 8-site composite pop (CEU-CHB)-CpG marker clearly separated European and Chinese samples with respect to their population affiliation. What is interesting, a better classification was obtained in peripheral blood samples than in LCL material (see Fig. 6).

To better characterize our composite pop (CEU-CHB)-CpG marker, we analyzed the genomic location of the differentially methylated CpGs. The vast majority of CpGs targeted by PyroAssays in this study were located outside of the, presumably evolutionary-conserved, CpG islands (see Table 1). CpG sites targeted by PyroAssays 5 and 8 were located in the shore regions (~ 2 kb from CpG islands, as defined by Illumina) of *Septin8* and *FLJ49/FLJ46906* genes, respectively. CpG sites targeted by PyroAssay 10 were situated in the body of *AGO3* gene. The only sites located in CpG islands (of *SHF* and *RREB1* genes, respectively) were those targeted by PyroAssays 6 and 9. Our results therefore concord with other studies, which have indicated that inter-population differences in DNA methylation level are enriched outside CpG islands and are concentrated in regions flanking the islands (shores, shelfs) or in gene body regions [42, 43].

Genes, in which our pop-CpGs reside, are involved in various biological processes: apoptosis regulation (*SHF*), expression regulation (*FLJ49/FLJ46906*), RNA interference (*AGO3*); or participate in distinct biological functions: transcription factor (*RRB1*), nucleotide binding protein (*SEPT8*). The biological relevance of the level of individual CpG sites methylation is still disputable [50]. However, it has been postulated that CpG sites located adjacent to functional genomics areas (CpG islands and/or shores) and representing similar methylation pattern due to potential effect on the chromatin structure, may play an important biological role [21]. In search for a putative long-range co-methylation, we examined five of the studied CpGs (cg08979191 chr5:132113734, cg04036182 chr15:45458818, cg18136963 chr6:139013146, cg07207043 chr6:7051497, cg23669876 chr1: 36489276). Methylation status of the neighboring CpG sites, located 200 bp up- and downstream from the pop-CpGs (co-methylation), was examined in samples from both populations (results in Additional file 5), using data from our HM450K array study ([40], data accessible through GEO Series accession number: GSE73901). Four of five pop-CpGs, cg08979191 (chr5:132113734), cg04036182 (chr15:45458818), cg18136963 (chr6:139013146), cg07207043 (chr6:7051497), had other Illumina-targeted CpGs in their vicinity (see Additional file 5). CpG sites located as far as 200 bp down- or upstream of two of the “core” CpGs (cg08979191, chr5:132113734 and cg18136963, chr6:139013146), displayed statistically significant inter-population differences in the level of methylation (IM_av__diffI in the range 0.8–2.5) (for details see Additional file 5). Importantly, the “direction” of these differences was the same as in the “core” cg08979191 and cg18136963 (the reduced level of methylation in individuals of Chinese in comparison to European ancestry. All the co-methylated CpG sites were located in the shore regions flanking CpG islands, of *SEPT8* and *FLJ49/FLJ46906* genes, respectively. A highly correlated methylation level of CpG sites separated by 200 bp suggests that cg08979191 (chr5:132113734) and cg18136963 (chr6:139013146) represent the methylation status of a longer region; this would be similar to the effect of linkage disequilibrium between SNPs in the human genome. However, it has to be kept in mind that Illumina HM450K array probes target a relatively small proportion of CpG sites in the human genome. A much larger number of neighboring CpG sites are present at the closer distance to these and the remaining pop-CpGs in our study; to examine methylation status of these sites, techniques addressing the whole genome should be employed, e.g. NGS technology.

DNA methylation constitutes an epigenetic switch in gene expression regulation [19, 20, 36, 51]. The relation between gene promotor methylation status and transcriptional regulation is well known and widely studied (e.g. [20, 36, 52]. However, recent studies also indicated more complex relation among DNA methylation status of CpG located in gene body regions, and/or intragenic sites and gene expression [51, 53, 54]. To determine, whether differentially methylated CpGs in our study reflected population differences in gene expression status, we integrated DNA methylation and gene expression data obtained from our previous studies performed on the same set of B-lymphocyte cell lines and conducted on two micorarrays systems: HM450K array and HumanHT-12v4 Expression BeadChip Kit expression array. All candidate pop-CpGs listed in Table 1 were subjected to Pearson correlation analysis. The results clearly demonstrate that among 14 candidate pop-CpGs, only one **cg24861686** (1_CpG1, chr8:11418058) located in the body of *BLK* gene, showed positive correlation between the gene expression and the methylation status. Such a positive correlation observed between the methylation status of a CpG localized in the gene body and the gene expression, was also observed in other studies (e.g. [36, 51, 55]). Among four others CpG sites located in *BLK* gene and tested in HM450K array, two: cg21701351 (chr8:11374774) and cg15685006 (chr8:11413044) were rejected from further analysis due to the presence of confounding features (SNPs/indels under probe and multi-site mapping). For two others: cg21497594, (chr8:11366745) and cg21175976 (chr8:11421338), positioned in 5’UTR region and gene body, respectively, no statistically significant differences in the methylation level between study populations were identified (q > 0.05). Therefore, neither cg21497594 nor cg21175976 were subjected to Pearson correlation analysis.

These results are not surprising, since the regulation of gene expression is a complex process involving e.g. transcription factors, histone modification, non-coding RNA regulation [51, 56–58]. A straight methylation-expression correlation is rarely observed, or is noted exclusively in individual genes [51]. In conclusion, the biological meaning of the differential methylation status observed in the analyzed populations remains to be elucidated.

A relationship between the genome and the methylome, as well as an association of DNA methylation with the gene expression regulation, are frequently discussed in the literature. There is ample literature indicating that 2/3 of methylation variability among population can be traced back to genetic ancestry ([15, 20, 36, 37, 43]. Therefore, to investigate the relationship between our population- differentiating CpG sites and the genetic background, we performed an in silico analysis of the genetic variability in the region ±10 kb around 14 selected pop-CpGs. For all tested pop-CpGs, the analysis showed the presence of a number of SNPs with Fst values in the range 0.00002–0.79 in 20 kb region. Selected SNPs with the highest CEU-CHB Fst values (0.28–0.79) are shown in Additional file 6. Our analysis suggest that inter-population differences in the methylation level could be due to the genetic variability of the analyzed populations. However, to draw conclusions regarding correlations between our population-differentiating CpG sites and individual SNPs, detailed similar analysis is required with respect to using the genetic data of individual B-lymphocyte cell lines used in this project would be necessary.

## Conclusions

Our results showed that even a small set of carefully selected differentially methylated CpGs (pop-CpGs), may be used to distinguish European and Chinese populations. Importantly, this composite pop (CEU-CHB)-CpG marker performs well in both lymphoblastoid cell lines and in non-homogenous blood samples regardless of a gender. The performance of our composite marker, estimated using different classification methods, was reasonably high for the limited number of examined samples, although this may change (either decrease or increase) when a larger number of sample are analyzed. Also, further studies using samples from other population groups need to be carried out.

The current knowledge regarding relation between epigenetics and environmental factors, as well as a trans-generation inheritance of methylation pattern (e.g. [36, 59]), is still limited. Nevertheless, it seems that discrimination between populations and inference of population origin of a sample, based on DNA methylation markers, is feasible and may add a new, additional dimension to medical and forensic casework, as earlier postulated [1, 60].

## Methods

### DNA samples

DNA samples from unrelated, healthy adult males and females representing European ancestry (mean age 38 years SD ± 10.3 years) and Chinese populations from Bejing with an exception of few samples from Japan (further referred to as CEU and CHB, respectively) (for details see Additional file 7), were isolated either from commercially available B-lymphocyte cell lines (Coriell Cell Repositories) or from samples of peripheral blood (CEU *n* = 20, CHB *n* = 20).

Both B-lymphocyte cell lines and peripheral blood samples used in this study underwent identical procedures including: DNA isolation (QIAamp DNA Blood Mini Kit, Qiagen), evaluation of its purity (Qubit, DSDNA H5 Assay Kit, Life Technology), and bisulfite treatment (EZ DNA Methylation-Gold Kit. Zymo Research). Five hundred ng of purified DNA from B-lymphocyte cell lines (*n* = 90), and peripheral blood (*n* = 40) was converted with bisulfite solution using EZ DNA Methylation–GoldTM Kit (Zymo Research, Germany), according to the manufacturer’s protocol.

### Study design

The study consisted of four main phases: selection of candidate pop-CpGs, two-step validation, and statistical tests (Fig. 1).

#### Selection of candidate pop-CpGs: identification of differentially metylated CpG sites based on *Human Methylation 450 BeadChip Array*

B-lymphocyte cell lines from CEU (*n* = 18) and CHB (*n* = 18) were examined on *Illumina Infinium HumanMethylation 450 BeadChip Array* (further referred to as HM450K array), according to the manufacturer-specified procedure. All analytical procedures, such as microarray technical quality evaluation, as well as statistical approach implemented in microarray data analysis, have been presented in detail in our previous publication see [40].

#### Technical validation: pyrosequencing assay design and optimization

Technical validation step was performed in a subset of B-lymphocyte cell lines previously analyzed by HM450K array. Pyrosequencing assays (further referred as PyroAssays) were designed to validate candidate pop-CpG sites preselected in HM450K array experiment for which effective PyroAssays could be designed (Assay score in PyroMark Assay Design Software ≥75, no CpGs under PyroAssay primers); in some cases, PyroAssays covered additional CpGs located in the close proximity (less than 25 bp upstream or downstream) of the selected candidate pop-CpGs (see Table 2 in Result section).

Wherever possible, PyroAssays were designed to analyze CpGs on the same DNA strand as in the microarray experiment, to eliminate possible differences in the CpG methylation status depending on the DNA strands (the only exceptions were PyroAssays 2, 4, 7) (for details see Additional file 8).

PCR reaction conditions (PCR program and further sample workflow) are available in Additional file 8. Primers for PyroAssays were designed using PyroMark Assay Design Software 2.0.1.15 (Qiagen). Only those PyroAssays, for which specific PCR products were obtained for both bisulfite converted study samples and for methylated/unmethylated controls, were used in further analyses (see Additional file 1).

The quality of methylation results collected from pyrosequencing reactions was assessed based on a series of dilution curves obtained for all the PyroAssays (see Additional file 9).

#### Biological validation: pyrosequencing assays in independent samples

CpGs that passed technical validation were further tested in two steps. In the first one, PyroAssays were examined in an independent set of B-lymphocyte cell lines from both populations (CEU *n* = 35; CHB *n* = 35); in the second step, PyroAssays were tested in peripheral blood samples (CEU *n* = 20; CHB *n* = 20) (see Fig. 1).

The same technical conditions (initial sample preparation, PCR reaction, Pyrosequencing process) were applied in both biological validation steps (see Additional files: 1 and 8).

#### Statistical analysis

Selection of the best non-redundant pop-CpGs from among those that passed technical and biological validation steps was conducted using beta regression test from the betareg Bioconductor package [61], with Benjamini Hochberg multiple testing correction.

The selected set of CpGs was then examined for its population-discriminating potential. Sample classification was conducted using three methods: support vector machines (SVM) with linear kernel, random forest (RF) and linear discriminant analysis (LDA). Prior to the classification process, correlated CpGs were removed; it was done by retaining only those with the lowest, adjusted *p*-values in the beta regression test for the technical validation dataset (20 samples), which was also used to select the best classification method. The predictive ability of the selected set of pop-CpGs was assessed using each of the classification methods, with 10-fold cross-validation, repeated 1000 times. In all cases classification was conducted using all possible combinations of 1 to 8 CpGs identified as differentially methylated.

The best classification method in terms of AUC (area under ROC curve) was than validated using two independent datasets from 48 B-lymphocyte cell lines and 40 total blood samples; all of the datasets were balanced (equal number of CEU and CHB samples). Classification was conducted in R with caret library and plotROC and ggplot2 used for visualization purposes.

Principal Component Analysys (PCA) was carried out in R using prcomp function from the stats package and visualized with the ggbiplot library.

In silico analysis of the genetic variability in the region ±10 kb around 14 selected pop-CpGs was carried out in R using Pegas package. Genomic data for a representative group of samples from both study populations (*n* = 198 CEU and *n* = 206 CHB) was obtained from 1000 Genomes database.

## Supplementary information

**Additional file 1.** Pyrosequencing procedures.

**Additional file 2.** A results of 3 classifiers cross-validation.

**Additional file 3.** A results of SVM classification performed on Male and Female B-lymphocyte cell lines obtained from GEO database (GSE36369).

**Additional file 4.** Biological validation of 8-point composite pop (CEU-CHB)-diff-met marker performed in blood samples.

**Additional file 5.** Comethylation results of Pyrosequencing Assays that underwent biological validation.

**Additional file 6.** A list of pop-CpGs and selected SNPs showing the highest CEU-CHB Fst value.

**Additional file 7.** A list of B-cell lines used in Illumina Microarray analysis and Pyrosequencing study.

**Additional file 8.** Pyrosequencig Assay designing and reaction optimization.

**Additional file 9.** Evaluation of PyroAssays sensitivity.

## Data Availability

The datasets generated and/or analyzed during the current study are available in GEO database with accession number GSE73901. The genomic sequences surrounding the study pop-CpGs were obtained from USCS Genome Browser database (https://genome.ucsc.edu/) based on genomic CpG site location (see main text). For an in silico analysis we used data from GEO database (GSE36369) and from 1000 Genome Project (https://www.internationalgenome.org). All of the datasets supporting the results of this article are included within the article and its Additional files.

## Abbreviations

CEU: Utah residents (CEPH) with Northern and Western European ancestry
CHB: Han Chinese in Beijing, China
HM450K array: *Illumina Infinium Human Methylation 450 BeadChip Array*
pop-CpGs: A set of CpG sites differentiating European and Chinese population based on methylation level differences
PyroAssays: Pyrosequencing assays
