# Supplementary material for: Discrimination between human populations using a small number of differentially methylated CpG sites: a preliminary study using lymphoblastoid cell lines and peripheral blood samples of European and Chinese origin
Source: BMC Genomics. 2020 Oct 12;21:706. doi: 10.1186/s12864-020-07092-x (PMC7549247; doi:10.1186/s12864-020-07092-x)
Supplement: Supplementary file 1 — Additional file 1. Pyrosequencing procedures. [file 12864_2020_7092_MOESM1_ESM.docx]

**Additional file 1:** Pyrosequencing procedures

10 PCR products were mixed with binding buffer (Qiagen, Hilden, Germany) and sepharose coated with streptavidin (GE Healthcare, Chicago, USA), shaked for 10 min and cleaned on vacuum pump station in the following buffers: 70% EtOH, 0.2% NaOH, and washing buffer (Qiagen, Hilden, Germany). Single-strand amplicons were then mixed with annealing buffer (Qiagen, Hilden, Germany), the sequencing primer (0.4 μM), and then heated for 2 min at 85°C and cooled for primer **hybridization**. Pyrosequencing was performed using the PyroMark Q24 (Qiagen, Hilden, Germany) and the results analyzed using the PyroMark Q24 (2.0.6 Qiagen software), which automatically calculates the G:A “−” strand (de facto mC:C) ratio at the CpG sites.

Each pyrosequencing run was accompanied by fully methylated DNA sample (MET) (Cells-to-CpG™ Methylated & Unmethylated gDNA Control Kit, Life Technology) and unmethylated (UMET) control. The Unmethylated control was prepared using the REPLI-g UltraFast Mini Kit (Sigma-Aldrich, Qiagen) from DNA isolated from B-lympocyte cell lines according to manufacturer procedure. The results for each analyzed sample were visualized as value bars of DNA methylation level in each CG repeat separately.

PyroAssays 4 and 7 were excluded from further studies, as a consequence of technical obstacles during PyroAssay optimalization process: in PyroAssay 4, no reproducible results were obtained in PCR (lack of MET and/or UMET controls amplification) and in PyroAssay 7, pyrosequencing reaction resulted in poor quality data (wide, low peaks, additional nucleotides visible in pyrogram). In PyroAssay 3, reliable measurement of the methylation level of cg00862290 was impossible due to the presence of a T-stretch located directly before the studied CpG; as a result, only two neighboring CpG sites located in the close vicinity to the candidate pop- CpG were examined.
