# Supplementary material for: Discrimination between human populations using a small number of differentially methylated CpG sites: a preliminary study using lymphoblastoid cell lines and peripheral blood samples of European and Chinese origin
Source: BMC Genomics. 2020 Oct 12;21:706. doi: 10.1186/s12864-020-07092-x (PMC7549247; doi:10.1186/s12864-020-07092-x)
Supplement: Supplementary file 2 — Additional file 2. A results of 3 classifiers cross-validation. [file 12864_2020_7092_MOESM2_ESM.docx]

**Additional file 2:**  A results of 3 classifiers cross-validation.

| **Classifier type** | **Precision**  **B-cell lines (Biological validation)**  **(n=24 CEU; n=24 CHB)** | **Precision**  **Blood Samples**  **(n=20 CEU; n=20 CHB)** | **AUC** |
| --- | --- | --- | --- |
| SVM | 0.854 | 0.850 | 0.996 |
| LDA | 0.8130 | 0.800 | 0.912 |
| RF | 0.771 | 0.600 | 0.946 |
