# Supplementary material for: Discrimination between human populations using a small number of differentially methylated CpG sites: a preliminary study using lymphoblastoid cell lines and peripheral blood samples of European and Chinese origin
Source: BMC Genomics. 2020 Oct 12;21:706. doi: 10.1186/s12864-020-07092-x (PMC7549247; doi:10.1186/s12864-020-07092-x)
Supplement: Supplementary file 3 — Additional file 3. A results of SVM classification performed on Male and Female B-lymphocyte cell lines obtained from GEO database (GSE36369). [file 12864_2020_7092_MOESM3_ESM.docx]

**Additional file 3:** A results of SVM classification performed on Male and Female B-lymphocyte cell lines obtained from GEO database (GSE36369).

| **Group** | **Precision** | **B-lymphocyte cell lines**  **n** |
| --- | --- | --- |
| Male | 0.899 | 93 |
| Female | 0.903 | 99 |
| All | 0.901 | 192 |
