# Supplementary material for: Discrimination between human populations using a small number of differentially methylated CpG sites: a preliminary study using lymphoblastoid cell lines and peripheral blood samples of European and Chinese origin
Source: BMC Genomics. 2020 Oct 12;21:706. doi: 10.1186/s12864-020-07092-x (PMC7549247; doi:10.1186/s12864-020-07092-x)
Supplement: Supplementary file 4 — Additional file 4. Biological validation of 8-point composite pop (CEU-CHB)-diff-met marker performed in blood samples. [file 12864_2020_7092_MOESM4_ESM.docx]

**Additional file4:** Biological validation of 8-point composite pop(CEU-CHB)-diff-met marker performed in blood samples.

Male and Female blood samples from both populations (CEU n=96 and CHB n=96) obtained from GEO database (GSE36369) were tested. Green – CEU population; blue – CHB population. Dots represent methylation level in the individual samples. Box plots denote mean value (lines inside the boxes) and standard deviation. Statistically significant (p<0.05) population differences in the methylation level are marked in red.

**
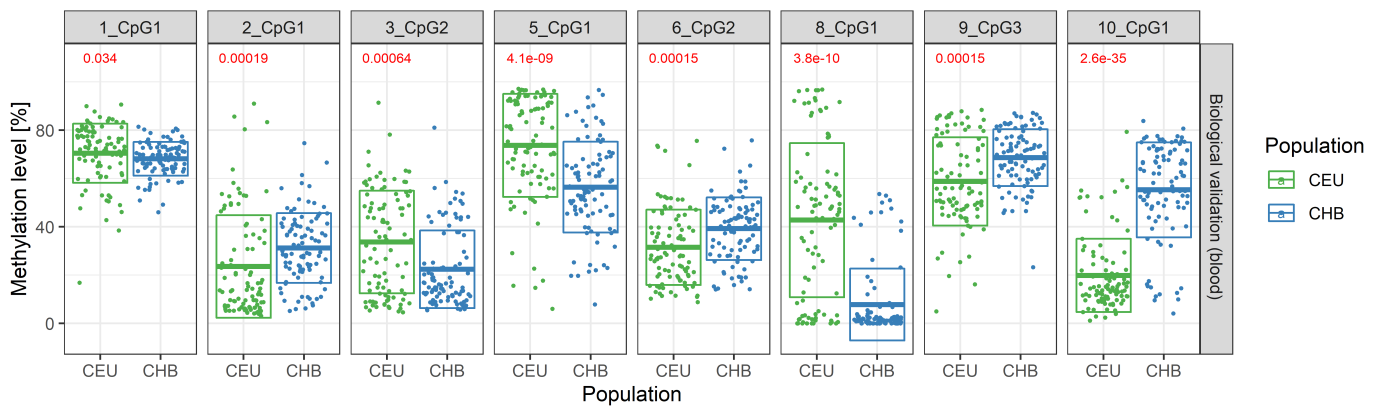
**
