# Supplementary material for: Discrimination between human populations using a small number of differentially methylated CpG sites: a preliminary study using lymphoblastoid cell lines and peripheral blood samples of European and Chinese origin
Source: BMC Genomics. 2020 Oct 12;21:706. doi: 10.1186/s12864-020-07092-x (PMC7549247; doi:10.1186/s12864-020-07092-x)
Supplement: Supplementary file 5 — Additional file 5. Comethylation results of Pyrosequencing Assays that underwent biological validation. [file 12864_2020_7092_MOESM5_ESM.docx]

| **Assay nb** | **450k-tagCpG name in Assay** | **IM_diff_I** | **Biological validation status** | **neighboring Illumina CpG to 450k-tagCpG**  **(200 bp down)** | **p-val** | **IM_diff_I neighboring CpG** | **neighboring Illumina CpG to 450k-tagCpG**  **(200 bp up-)** | **p-val** | **IM_diff_I neighboring CpG** |
| --- | --- | --- | --- | --- | --- | --- | --- | --- | --- |
|  |  |  |  |  |  |  |  |  |  |
| 5 | cg08979191* | 1.875 | validated | **cg13334727** | 0.0360 | 0.793 | **cg25212993** | 0.0021 | 0.996 |
| 5 |  |  |  | cg23077575 | p>0.05 |  | **cg06941635** | 0.0005 | 1.105 |
| 5 |  |  |  | **cg19518672** | 0.0440 | 0.914 | **cg01444808** | 0.0083 | 0.876 |
| 5 |  |  |  | **cg27309611** | 0.0047 | 0.882 |  |  |  |
| 6 | cg04036182 | 1.451 | valideted | **-** | **-** | **-** | cg17366378 | p>0.05 |  |
| 8 | cg18136963* | 2.950 | validated | **cg06864789** | 0.0001 | 2.461 | **-** | **-** | **-** |
| 8 |  |  |  | **cg25399239** | 0.0001 | 2.491 |  |  |  |
| 9 | cg07207043 | 1.534 | validated | cg03475293 | p>0.05 |  | **-** | **-** | **-** |
| 10 | cg23669876 | 2.355 | validated | **-** | **-** | **-** | **-** | **-** | **-** |

Additional file 5: Comethylation results of Pyrosequencing Assays that underwent biological validation.

With * marked PyroAssays with neighboring CpGs with statistically significant differences in the level of methylation between populations
