# Supplementary material for: Discrimination between human populations using a small number of differentially methylated CpG sites: a preliminary study using lymphoblastoid cell lines and peripheral blood samples of European and Chinese origin
Source: BMC Genomics. 2020 Oct 12;21:706. doi: 10.1186/s12864-020-07092-x (PMC7549247; doi:10.1186/s12864-020-07092-x)
Supplement: Supplementary file 6 — Additional file 6. A list of pop-CpGs and selected SNPs showing the highest CEU-CHB Fst value. [file 12864_2020_7092_MOESM6_ESM.docx]

**Additional file 6:** A list of pop-CpGs and selected SNPs showing the highest CEU-CHB Fst value.

Pop-CpGs selected for pyrosequencing validation are bolded. **DISTANCE**- SNP distance from pop-CpG; **CHROM**- SNP position on chromosome; **REF**- ancestral allele, **ALT**- derived allele; **CEU_ref_num**- number of individuals with ancestral allele; **CEU_alt_num**- number of individuals with derived allele; **CEU_alt_AF -** CEU_alt_num/( CEU_alt_num+ CEU_alt_num); **Fst-** Fst value

| **cpgID** | **ID** | **DISTANCE** | **CHROM** | **POS** | **REF** | **ALT** | **CEU_ref_num** | **CEU_alt_num** | **CEU_alt_AF** | **CHB_ref_num** | **CHB_alt_num** | **CHB_alt_AF** | **Fst** |
| --- | --- | --- | --- | --- | --- | --- | --- | --- | --- | --- | --- | --- | --- |
| cg00031303 | rs1679295 | -7930 | 3 | 1.96E+08 | C | G | 169 | 29 | 0.146465 | 46 | 160 | 0.776699 | 0.565889 |
| **cg00862290** | rs2864412 | -3632 | 3 | 1.79E+08 | T | A | 106 | 92 | 0.464646 | 21 | 185 | 0.898058 | 0.351355 |
| **cg03140118** | rs11264060 | 5186 | 1 | 37944506 | G | A | 132 | 66 | 0.333333 | 49 | 157 | 0.762136 | 0.306603 |
| cg03585734 | rs6703580 | 295 | 1 | 15599160 | G | T | 93 | 105 | 0.530303 | 14 | 192 | 0.932039 | 0.336717 |
| **cg04036182** | rs1706815 | 117 | 15 | 45458935 | C | G | 87 | 111 | 0.560606 | 202 | 4 | 0.019417 | 0.524199 |
| **cg07207043** | rs592377 | 9405 | 6 | 7060902 | C | T | 39 | 159 | 0.80303 | 201 | 5 | 0.024272 | 0.76964 |
| **cg07904028** | rs4689403 | -737 | 4 | 6327771 | T | C | 76 | 122 | 0.616162 | 0 | 206 | 1 | 0.382464 |
| **cg08979191** | rs30512 | 5423 | 5 | 1.32E+08 | C | T | 173 | 25 | 0.126263 | 50 | 156 | 0.757282 | 0.569685 |
| cg09972454 | rs11646144 | -5429 | 16 | 15077659 | C | G | 139 | 59 | 0.29798 | 205 | 1 | 0.004854 | 0.283284 |
| **cg18136963** | rs71667027 | 9848 | 6 | 1.39E+08 | T | TTA | 152 | 46 | 0.232323 | 48 | 158 | 0.76699 | 0.439072 |
| **cg23669876** | rs2336659 | -4995 | 1 | 36484281 | C | A | 198 | 0 | 0 | 54 | 152 | 0.737864 | 0.731394 |
| cg24037715 | rs10637549 | -9106 | 14 | 35194862 | C | CTTT | 192 | 6 | 0.030303 | 34 | 172 | 0.834951 | 0.790606 |
| **cg24861686** | rs13248757 | 3263 | 8 | 11421321 | T | C | 153 | 45 | 0.227273 | 14 | 192 | 0.932039 | 0.67399 |
| **cg26367031** | rs2864412 | -3406 | 3 | 1.79E+08 | T | A | 106 | 92 | 0.464646 | 21 | 185 | 0.898058 | 0.351355 |
