# Supplementary material for: Discrimination between human populations using a small number of differentially methylated CpG sites: a preliminary study using lymphoblastoid cell lines and peripheral blood samples of European and Chinese origin
Source: BMC Genomics. 2020 Oct 12;21:706. doi: 10.1186/s12864-020-07092-x (PMC7549247; doi:10.1186/s12864-020-07092-x)
Supplement: Supplementary file 7 — Additional file 7. A list of B-cell lines used in Illumina Microarray analysis and Pyrosequencing study. [file 12864_2020_7092_MOESM7_ESM.docx]

Additional file 7: A list of B-cell lines used in Illumina Microarray analysis and Pyrosequencing study.

| **NB** | **SAMPLE NAME** | **CORIELL NUMBER** | **STUDY** | **ETHNICITY** | **SEX** |
| --- | --- | --- | --- | --- | --- |
| 1 | AG18183 | AG18183 | Microarray/Pyrosequencing | Caucasian, Georgia collection | Male |
| 2 | AG18193 | AG18193 | Microarray/Pyrosequencing | Caucasian, Georgia collection | Male |
| 3 | AG18219 | AG18219 | Microarray/Pyrosequencing | Caucasian, Georgia collection | Male |
| 4 | AG18236 | AG18236 | Microarray/Pyrosequencing | Caucasian, Georgia collection | Male |
| 5 | AG18250 | AG18250 | Microarray/Pyrosequencing | Caucasian, Georgia collection | Male |
| 6 | AG19409 | AG19409 | Microarray/Pyrosequencing | Caucasian, Georgia collection | Male |
| 7 | AG19412 | AG19412 | Microarray/Pyrosequencing | Caucasian, Georgia collection | Male |
| 8 | AG19414 | AG19414 | Microarray/Pyrosequencing | Caucasian, Georgia collection | Male |
| 9 | AG19505 | AG19505 | Microarray/Pyrosequencing | Caucasian, Georgia collection | Male |
| 10 | AG19579 | AG19579 | Microarray/Pyrosequencing | Caucasian, Georgia collection | Male |
| 11 | GM18608 | GM18608 | Microarray/Pyrosequencing | Han Chinese from Beijing | Male |
| 12 | GM18609 | GM18609 | Microarray/Pyrosequencing | Han Chinese from Beijing | Male |
| 13 | GM18611 | GM18611 | Microarray/Pyrosequencing | Han Chinese from Beijing | Male |
| 14 | GM18612 | GM18612 | Microarray/Pyrosequencing | Han Chinese from Beijing | Male |
| 15 | GM18620 | GM18620 | Microarray/Pyrosequencing | Han Chinese from Beijing | Male |
| 16 | GM18621 | GM18621 | Microarray/Pyrosequencing | Han Chinese from Beijing | Male |
| 17 | GM18622 | GM18622 | Microarray/Pyrosequencing | Han Chinese from Beijing | Male |
| 18 | GM18623 | GM18623 | Microarray/Pyrosequencing | Han Chinese from Beijing | Male |
| 19 | GM18635 | GM18635 | Microarray/Pyrosequencing | Han Chinese from Beijing | Male |
| 20 | GM18643 | GM18643 | Microarray/Pyrosequencing | Han Chinese from Beijing | Male |
| 21 | AG1 | AG15200 | Pyrosequencing | Hungarian | Male |
| 22 | AG2 | AG15202 | Pyrosequencing | Hungarian | Male |
| 23 | AG3 | AG15205 | Pyrosequencing | Hungarian | Male |
| 24 | AG4 | AG15206 | Pyrosequencing | Hungarian | Male |
| 25 | AG5 | AG15208 | Pyrosequencing | Hungarian | Male |
| 26 | AG6 | AG17003 | Pyrosequencing | North European | Male |
| 27 | AG7 | AG17007 | Pyrosequencing | North European | Male |
| 28 | AG8 | AG17008 | Pyrosequencing | North European | Male |
| 29 | AG9 | AG17010 | Pyrosequencing | North European | Male |
| 30 | AG10 | AG17324 | Pyrosequencing | Italian | Male |
| 31 | AG11 | AG17325 | Pyrosequencing | Italian | Male |
| 32 | AG12 | AG17326 | Pyrosequencing | Italian | Male |
| 33 | AG13 | GM15724 | Pyrosequencing | Czech | Male |
| 34 | AG14 | GM15725 | Pyrosequencing | Czech | Male |
| 35 | AG15 | GM15727 | Pyrosequencing | Czech | Male |
| 36 | AG16 | GM15728 | Pyrosequencing | Czech | Male |
| 37 | AG17 | AG18190 | Pyrosequencing | Caucasian, Georgia collection | Male |
| 38 | AG18 | AG18192 | Pyrosequencing | Caucasian, Georgia collection | Male |
| 39 | AG19 | AG18224 | Pyrosequencing | Caucasian, Georgia collection | Male |
| 40 | AG20 | AG18245 | Pyrosequencing | Caucasian, Georgia collection | Male |
| 41 | AG41 | AG18189 | Pyrosequencing | Caucasian, Georgia collection | Male |
| 42 | AG42 | AG18215 | Pyrosequencing | Caucasian, Georgia collection | Female |
| 43 | AG43 | AG18237 | Pyrosequencing | Caucasian, Georgia collection | Female |
| 44 | AG44 | AG18238 | Pyrosequencing | Caucasian, Georgia collection | Female |
| 45 | AG45 | AG18244 | Pyrosequencing | Caucasian, Georgia collection | Male |
| 46 | AG46 | AG18257 | Pyrosequencing | Caucasian, Georgia collection | Male |
| 47 | AG47 | AG18271 | Pyrosequencing | Caucasian, Georgia collection | Male |
| 48 | AG48 | AG18300 | Pyrosequencing | Caucasian, Georgia collection | Male |
| 49 | AG49 | AG19416 | Pyrosequencing | Caucasian, Georgia collection | Male |
| 50 | AG50 | AG19425 | Pyrosequencing | Caucasian, Georgia collection | Female |
| 51 | AG51 | AG19495 | Pyrosequencing | Caucasian, Georgia collection | Male |
| 52 | AG52 | AG19496 | Pyrosequencing | Caucasian, Georgia collection | Male |
| 53 | AG53 | AG19497 | Pyrosequencing | Caucasian, Georgia collection | Male |
| 54 | AG54 | AG19508 | Pyrosequencing | Caucasian, Georgia collection | Female |
| 55 | AG55 | AG19520 | Pyrosequencing | Caucasian, Georgia collection | Male |
| 56 | GM21 | GM17965 | Pyrosequencing | Chinese in Denver | Male |
| 57 | GM22 | GM17967 | Pyrosequencing | Chinese in Denver | Male |
| 58 | GM23 | GM17969 | Pyrosequencing | Chinese in Denver | Male |
| 59 | GM24 | GM17972 | Pyrosequencing | Chinese in Denver | Male |
| 60 | GM25 | GM17973 | Pyrosequencing | Chinese in Denver | Male |
| 61 | GM26 | GM17974 | Pyrosequencing | Chinese in Denver | Male |
| 62 | GM27 | GM17975 | Pyrosequencing | Chinese in Denver | Male |
| 63 | GM28 | GM17976 | Pyrosequencing | Chinese in Denver | Male |
| 64 | GM29 | GM17979 | Pyrosequencing | Chinese in Denver | Male |
| 65 | GM30 | GM17980 | Pyrosequencing | Chinese in Denver | Male |
| 66 | GM31 | GM17983 | Pyrosequencing | Chinese in Denver | Male |
| 67 | GM32 | GM17986 | Pyrosequencing | Chinese in Denver | Male |
| 68 | GM33 | GM18546 | Pyrosequencing | Han Chinese from Beijing | Male |
| 69 | GM34 | GM18557 | Pyrosequencing | Han Chinese from Beijing | Male |
| 70 | GM35 | GM18603 | Pyrosequencing | Han Chinese from Beijing | Male |
| 71 | GM36 | GM18647 | Pyrosequencing | Han Chinese from Beijing | Male |
| 72 | GM37 | GM18940 | Pyrosequencing | Japanese, Tokyo | Male |
| 73 | GM38 | GM18945 | Pyrosequencing | Japanese, Tokyo | Male |
| 74 | GM39 | GM18948 | Pyrosequencing | Japanese, Tokyo | Male |
| 75 | GM40 | GM18953 | Pyrosequencing | Japanese, Tokyo | Male |
| 76 | GM62 | GM18524 | Pyrosequencing | Han Chinese from Beijing | Male |
| 77 | GM63 | GM18526 | Pyrosequencing | Han Chinese from Beijing | Female |
| 78 | GM64 | GM18529 | Pyrosequencing | Han Chinese from Beijing | Female |
| 79 | GM65 | GM18530 | Pyrosequencing | Han Chinese from Beijing | Male |
| 80 | GM66 | GM18534 | Pyrosequencing | Han Chinese from Beijing | Male |
| 81 | GM68 | GM18537 | Pyrosequencing | Han Chinese from Beijing | Female |
| 82 | GM69 | GM18542 | Pyrosequencing | Han Chinese from Beijing | Female |
| 83 | GM70 | GM18543 | Pyrosequencing | Han Chinese from Beijing | Male |
| 84 | GM71 | GM18548 | Pyrosequencing | Han Chinese from Beijing | Male |
| 85 | GM72 | GM18549 | Pyrosequencing | Han Chinese from Beijing | Male |
| 86 | GM73 | GM18558 | Pyrosequencing | Han Chinese from Beijing | Male |
| 87 | GM74 | GM18559 | Pyrosequencing | Han Chinese from Beijing | Male |
| 88 | GM75 | GM18561 | Pyrosequencing | Han Chinese from Beijing | Male |
| 89 | GM76 | GM18572 | Pyrosequencing | Han Chinese from Beijing | Male |
