# Supplementary material for: Discrimination between human populations using a small number of differentially methylated CpG sites: a preliminary study using lymphoblastoid cell lines and peripheral blood samples of European and Chinese origin
Source: BMC Genomics. 2020 Oct 12;21:706. doi: 10.1186/s12864-020-07092-x (PMC7549247; doi:10.1186/s12864-020-07092-x)
Supplement: Supplementary file 8 — Additional file 8. Pyrosequencig Assay designing and reaction optimization. [file 12864_2020_7092_MOESM8_ESM.docx]

Additional file 8: Pyrosequencig Assay designing and reaction optimization

PCR was performed using PyroMark PCR kit (Qiagen) and the reaction mixture (25 μl) composed of: 1x PyroMark Master Mix (contains HotStartTaq DNA Polymerase, 1x PyroMark PCR Buffer and dNTPs), 10 pmol of each primer, 1x CoralLoad Concentrate and 1 μl of converted DNA.

The PCR reaction program conditions are as follows: at 95°C for 15 min (initial heating), followed by 45 cycles: denaturation for 30 s at 94°C, primer annealing (temperatures available in Table below) for 30 s and extension in 72°C for 30 s, and the final extension in 72°C for 10 min.

PCR products were run on 1.5% agarose gel and were visualized under UV light.

| **Pyrosequencing Assays name** | **Location of CpG on the DNA strand** | **Primer name** | **Primer sequence 5'-3'** | **Assay score**  **In**  **PyroMark Assay Design Software** | **Primer Tm in PCR reaction** | **Amplicon size in PCR reaction (bp)** |
| --- | --- | --- | --- | --- | --- | --- |
| PyroAssay1 | F | 1_cg24861686_F | GTTAGGGATAGTAGTTGGTGTT | 78 | 60^o^C | 196 |
|  |  | 1_cg24861686_R-biot | CATAAATAAACCAACAAAATACAAACAT |  |  |  |
|  |  | 1_cg24861686_seq | AAGGTTTTATTTATTTAATTAATGG |  |  |  |
|  |  |  |  |  |  |  |
| PyroAssay 2 | R | 2_cg03140118_F | TAGTTTTTGTAGAATTTTTTAAGGGTAAT | 90 | 60^o^C | 119 |
|  |  | 2_cg03140118_R-biot | ATTTCCCAAATCAAACCTAATCC |  |  |  |
|  |  | 2_cg03140118_seq | AATATTAGTATAATGATTTTTGTT |  |  |  |
|  |  |  |  |  |  |  |
| PyroAssay 3 | F | 3_cg00862290_F | TTTAGTTTAAGGTTTATAAGAAGAGGAAA | 87 | 55^o^C | 98 |
|  |  | 3_cg00862290_R-biot | AAACCCATTATTTCAACTTACACTC |  |  |  |
|  |  | 3_cg00862290_seq | GGAAATGTTTTGTTTTGG |  |  |  |
|  |  |  |  |  |  |  |
| PyroAssay 4 | R | 4_cg07904028_F | AGGGTAGAATGTGGATTAGAGATTGAGA | 86 | 55^o^C | 186 |
|  |  | 4_cg07904028_R-biot | ATTCCTAATAACCCCCTCTAACAAT |  |  |  |
|  |  | 4_cg07904028_seq | AGAGTTGTTGTTTTTTTATAGT |  |  |  |
|  |  |  |  |  |  |  |
| PyroAssay 5 | F | 5_cg08979191_F | AGATTGTTAGTGAGAAGGATATAGAA | 84 | 60^o^C | 143 |
|  |  | 5_cg08979191_R-biot | CCACCATTATACAAAAAAAATAACCACTC |  |  |  |
|  |  | 5_cg08979191_seqS | GTTTTGAGTAGTGATATT |  |  |  |
|  |  |  |  |  |  |  |
| PyroAssay 6 | F | 6_cg04036182_F | GGGTAGGTTGGTTAGGATTTTT | 76 | 60^o^C | 190 |
|  |  | 6_cg04036182_R-biot | CCTACATACCACATATCCTCAATTTC |  |  |  |
|  |  | 6_cg04036182_seq | GATATGTTGGATAGGTATT |  |  |  |
|  |  |  |  |  |  |  |
| PyroAssay 7 | R | 7_cg26367031_F | TTGGAGAGGTGAGAATTTTAGGTAG | 79 | 60^o^C | 232 |
|  |  | 7_cg26367031_R-biot | TTAAACCTCAAAACTTTCCACAAAC |  |  |  |
|  |  | 7_cg26367031_seq | TTTTAGATGATTAAGAAGGAT |  |  |  |
|  |  |  |  |  |  |  |
| PyroAssay 8 | F | 8_cg18136963_F | GAAGAGAGGTGGTAAGTAGTTT | 82 | 55^o^C | 242 |
|  |  | 8_cg18136963_R-biot | AATCCCCTTATCTACTCCTTCT |  |  |  |
|  |  | 8_cg18136963_seq | GAGGTGGTAAGTAGTTTTA |  |  |  |
|  |  |  |  |  |  |  |
| PyroAssay 9 | R | 9_cg07207043_F-biot | GGAGAGTGTGAAGGTGATTT | 75 | 60^o^C | 146 |
|  |  | 9_cg07207043_R | CCCCACTAAAACTTCAAAATATCT |  |  |  |
|  |  | 9_cg07207043_seq | CAAAATATCTAAACTACATAAATTC |  |  |  |
|  |  |  |  |  |  |  |
| PyroAssay10 | R | 10_cg23669876_F | TGAATATGGTTTTAATAGTTGGTTGTAT | 85 | 55^o^C | 193 |
|  |  | 10_cg23669876_R-biot | CATCCTCAACAAAAAAATAAAAATCTTAAT |  |  |  |
|  |  | 10_cg23669876_seq | TGGTATAAGAATAGGTTATAGT |  |  |  |
