# Supplementary material for: Discrimination between human populations using a small number of differentially methylated CpG sites: a preliminary study using lymphoblastoid cell lines and peripheral blood samples of European and Chinese origin
Source: BMC Genomics. 2020 Oct 12;21:706. doi: 10.1186/s12864-020-07092-x (PMC7549247; doi:10.1186/s12864-020-07092-x)
Supplement: Supplementary file 9 — Additional file 9. Evaluation of PyroAssays sensitivity. [file 12864_2020_7092_MOESM9_ESM.docx]

**Additional file 9:** Evaluation of PyroAssays sensitivity

The quality of methylation results collected from pyrosequencing reactions was assessed based on a series of dilution curves (from 0 % to 100%, every 10%) obtained for all the PyroAssays.

A good reproducibility and sensitivity was obtained for all 8 PyroAssays (see an example of dilution curve for PyroAssay 5; the other are available upon request).

Figure S1. An example of dilution curve obtained for PyroAssay 5.
